# Noise Directivity Reconstruction Radiated from Unmanned Underwater Vehicle’s Propeller Using the Equivalent Source Method

**DOI:** 10.3390/s25051466

**Published:** 2025-02-27

**Authors:** Shuai Jiang, Liwen Tan, Ruichong Gu, Zilong Peng

**Affiliations:** School of Energy and Power Engineering, Jiangsu University of Science and Technology, Zhenjiang 212100, China; jiangshuai1201@163.com (S.J.); grc223@126.com (R.G.); zlp_just@sina.com (Z.P.)

**Keywords:** unmanned underwater vehicles, propeller noise, equivalent source method, acoustic radiation

## Abstract

Noise directivity reconstruction and prediction of noise levels at long ranges from such sources as unmanned underwater vehicles (UUVs) or aircraft are important practical problems. The equivalent source method can be used to reconstruct and predict the sound propagation of such directional complex volume sources in the far field. However, the selection of the elementary source configurations for the equivalent source method has a certain degree of blindness. In this paper, a method for selecting elementary source configurations was proposed, considering the correlation coefficients that exhibit a strong correlation with the directivity function. It is then applied to reconstruct the noise directivity pattern radiated from a real UUV. The results demonstrate that this method can achieve higher accuracy in reconstructing complex radiated sound sources using fewer elementary source configurations.

## 1. Introduction

With the increasing use of unmanned underwater vehicles (UUVs), the issue of underwater radiated noise has gained significant attention. At the same time, advances in computer technology have made numerical algorithms the primary approach for studying the acoustic radiation of complex underwater vibrating bodies. Currently, the most widely used methods are the Finite Element Method (FEM) [1] and the Boundary Element Method (BEM) [2]. The FEM requires discretizing the entire solution domain, which is usually limited to sound field calculations in finite domains. In contrast, the BEM reduces the dimensionality of the analysis and is more suitable for sound field calculations in infinite domains, offering advantages over the FEM [3]. However, the BEM has issues with handling singular integrals, which not only increase computational difficulty but also, if handled improperly, can affect the accuracy of the results [4].

To address this, Koopmann et al. proposed the Wave Superposition Method (WSM) [5], which is based on the idea of fitting the radiated acoustic field of a real source by superimposing the acoustic fields generated by a series of virtual equivalent sources, thus achieving sound field reconstruction and prediction. To improve efficiency, the unit integrals in the WSM are usually simplified to monopole point sources, which is known as the Equivalent Source Method (ESM) [6]. Compared with the BEM method, the ESM method has several advantages. First, ESM is a meshless method, utilizing simple point sources both inside and outside the surface. Second, it avoids numerical singularity, as the distance between the equivalent source points and the matching points is never zero. Third, the number of equivalent sources does not necessarily have to match the number of matching points, providing flexibility in balancing accuracy and computation time. By reducing the number of equivalent sources, the size of the linear system and computation time can be significantly reduced. Dunn et al. [7] proposed guidelines on selecting the number and position of equivalent sources, recommending a value of approximately $Sk^{2}$, where k is the reference wave number, and S represents the scattering surface area. Additionally, the ESM has been applied to inverse reconstruction problems to determine the geometry of actual noise sources or radiators [8,9].

To accommodate structures of arbitrary shapes and improve computational stability, equivalent sources are typically arranged uniformly on a closed virtual boundary that conforms to the actual boundary. Jeans et al. [10,11] pointed out that the use of a smooth closed surface as the configuration region for the virtual sources leads to non-unique solutions for the sound field at the characteristic frequencies of the corresponding problem on the virtual surface. Ochmann et al. [12] discussed that the non-uniqueness issue can be avoided by using layered or multi-point methods because the equivalent sources do not enclose the internal space. This can be achieved by positioning the equivalent sources along straight lines or selecting non-enclosed surfaces. Lu Jing et al. [13,14] proposed a complex radius vector wave superposition method, which only requires adding a small amount of complex virtual damping to the position radius vector of the virtual surface to obtain the unique solution in the entire frequency domain. However, subsequent research revealed significant limitations of this method, as it is only applicable to relatively regular and smooth boundary shapes. For boundaries of arbitrary shapes, this method imposes strict requirements on the selection of damping coefficients and may even face the risk of failure.

In computational methods for far-field sound propagation, omnidirectional point sources are typically assumed, such as ray tracing [15,16], fast field programs [17,18], and the parabolic equation method [19,20]. This assumption does not necessarily limit the application, as more complex sound sources can be represented by the spatial distribution of omnidirectional point sound sources. However, in many practical cases, due to the complexity of the sound sources, the sound source or its analytical expression as a point source is unknown. For example, UUVs or aircraft. These sources exhibit directional radiation, and the challenge in calculating far-field sound propagation for such sound sources lies in how to specify these directional sources in numerical propagation algorithms. For the far-field sound propagation problem of directional volume sources, Vecherin et al. [21,22] associated spherical harmonic functions with specific compact elementary source configurations, reproducing the given directional radiation patterns in the far field. They also discussed the impact of outliers and data incompleteness on this method through the study of actual aircraft-radiated noise. Compared with the traditional ESM, this method does not require the equivalent sources to be uniformly distributed on the actual boundary, conforming to the closed virtual boundary. Therefore, this method requires only a small number of equivalent sources and can be applied to more complex models. This method has been cited or directly applied in sound field calculations by some researchers, yielding good results [23,24,25]. However, this method has a degree of blindness when selecting elementary source configurations, and to achieve better results, a larger number of elementary source configurations are usually used.

Therefore, this paper introduces a method for selecting elementary source configurations of equivalent sources by introducing correlation coefficients, aiming to achieve better results with fewer elementary source configurations, and applies this method to the reconstruction of the propeller radiated noise from a UUV. First, the reconstruction of a constructed directivity function is performed to explore the general principles for selecting elementary source configurations. By introducing correlation coefficients, it is concluded that the elementary source configurations should be selected based on their strong correlation with the directivity function. Then, this method is applied to the reconstruction of the propeller radiation noise from a UUV in actual tests. Through both narrow and wide-angle reconstructions, the method is verified to achieve higher accuracy in reconstructing complex radiated sound sources using fewer elementary point source configurations. The structure of the paper is as follows: The structure of this paper is as follows: Section 2 introduces the equivalent source method used in this paper; Section 3 discusses the method for selecting elementary point source configurations. Section 4 provides a detailed explanation of the application of this method in the reconstruction of UUV propeller-radiated noise. Finally, Section 5 summarizes the research findings and suggests potential directions for future studies.

## 2. Equivalent Source Method

The methods for modeling directional sound sources in the far-field are described in detail in References [21,22]. This chapter provides a brief overview of this method.

### 2.1. Modeling Directional Sound Sources with ESM

A far-field radiation pattern in free space is represented by a directivity function $D\left(\theta ,\varphi \right)$, which is defined by the following equation:

(1) $$p\left(R\right)=D\left(\theta ,\varphi \right)\frac{\exp\left(ik_{0}R\right)}{R},$$

where p represents the complex acoustic pressure; R is the three-dimensional spatial vector that defines the position of the observation point. The directivity function $D\left(\theta ,\varphi \right)$ dependent on the spherical angles $\left(\theta ,\varphi \right)$; $k_{0}$ is the wave number, and the magnitude of the spatial vector is $R=\left|R\right|$. Equation (1), as an approximate solution to the Helmholtz equation, is valid in the far field for any complex sound sources located near the origin. More precisely, the far-field condition that the observation point R must satisfy is: $R\gg max\left\{\lambda ,d_{max},{d_{max}}^{2}/\lambda \right\}$, where λ is the wavelength, and $d_{max}$ is the maximum distance from the origin to any point of the source. For a point source that radiates omnidirectionally with amplitude A, the directivity function becomes D=A, and Equation (1) becomes an exact solution to the Helmholtz equation. ESM aims to reconstruct an equivalent distribution of compact point sources, determining their numbers, amplitudes, and locations so that they produce a given directivity function (the compactness condition: $k_{0}d_{max}\ll 1$).

ESM consists of three steps. First, the directivity function is decomposed in terms of spherical harmonics:

(2) $$D\left(\theta ,\varphi \right)=\overset{\infty }{\underset{l=0}{\sum }}\overset{l}{\underset{m=-l}{\sum }}c_{l}^{m}Y_{l}^{m}\left(\theta ,\varphi \right),$$

(3) $$Y_{l}^{m}\left(\theta ,\varphi \right)=\frac{1}{2}\sqrt{\frac{2l+1}{\pi }\frac{\left(l-\left|m\right|\right)!}{\left(l+\left|m\right|\right)!}}P_{l}^{\left|m\right|}\left(cos\theta \right)\exp\left(im\varphi \right),$$

where $Y_{l}^{m}$ represents the normalized spherical harmonic of order *l* and degree *m*; $P_{l}^{\left|m\right|}$ are the associated Legendre polynomials ($\left|m\right|\leq l$). The coefficients $c_{l}^{m}$ can be easily obtained using the orthonormality property of the spherical harmonics, with the result:

(4) $$c_{l}^{m}=\int_{0}^{2\pi }d\varphi \int_{0}^{\pi }D\left(\theta ,\varphi \right)Y_{l}^{*m}\sin\theta d\theta ,$$

where the asterisk indicates the complex conjugate. In practical applications, the infinite series on *l* is limited to some maximal value, ensuring that the original directional function is sufficiently consistent with its series representation defined by Equation (2). Such a criterion can be a normalized root-mean-square error (NRMSE) below a specified threshold. Although the azimuthal angle φ in Equation (3) is defined within the range [0,2π), it can sometimes be used within the interval $\left[-\pi ,\pi \right]$.

Second, each spherical harmonic function can be linked to a specific compact point source configuration, which will be called an elementary source configuration (the elementary source configurations corresponding to the first three orders of spherical harmonic functions are shown in Table 1). The radiation patterns of these elementary source configurations are equivalent to the corresponding spherical harmonic functions. It is important to note that the elementary source configurations only need to be determined once, after which they can be saved for the reconstruction of new directivity functions.

Third, once the required elementary source configurations are identified, their linear combinations can reconstruct the given directivity function, with the coefficients determined by Equation (3).

### 2.2. Application to the PE Algorithms

ESM can be applied with any propagation algorithm, including Parabolic Equations (PEs). However, when dealing with point sources moving horizontally from the origin, its application becomes more complex, as it requires defining separate PE initial fields at their horizontal coordinates and conducting individual PE calculations. To address this, we focus on two-dimensional PEs. By fixing ${\varphi =\varphi }_{0}$, we select a specific plane $\left(r,z\right)$, where *r* represents the horizontal distance, and *z* is the vertical distance. In Equation (1), $R=\sqrt{r^{2}+z^{2}}$ and the directivity function in free space $D\left(\theta ,\varphi_{0}\right)$ depends solely on the angle θ, allowing it to be represented using the vertical source configuration. This allows the sound field for different φ values to be considered separately. It is important to note that the source configurations may vary for different values of φ. For a point source moving horizontally from the origin, a method for converting the horizontal source configurations into vertical ones is described in Reference [21].

In the case when $\left|z\right|/r<1$, in the vicinity of θ≈π/2, the function sin⁡θ can be decomposed into a series of even-powered cosines with arbitrary accuracy. The precision required for the narrow-angle PE is obtained by decomposing sin⁡θ as: $\sin\theta =1-\frac{1}{2}\cos^{2}\theta$. The precision required for the wide-angle PE is obtained by decomposing sin⁡θ as: $\sin\theta =1-\frac{1}{2}\cos^{2}\theta -\frac{1}{8}\cos^{4}\theta$.

The θ-angle range and applicable scenarios for narrow-angle PE and wide-angle PE are listed in Table 2.

For example, the directivity function of a horizontal dipole aligned with the *x*-axis can be simulated using three vertically arranged point sources with narrow-angle PE or five sources for vertically arranged point sources with wide-angle PE.

In principle, any desired source directivity can be represented as a superposition of vertically oriented elementary source configurations. By determining the number of these source configurations and their corresponding coefficients as needed, the given directivity function can be reproduced with sufficient accuracy within the specified θ-range. Although Equation (4) may not be suitable in this case, a solution can be efficiently obtained using a least-squares formulation.

For a fixed-angle ${\varphi =\varphi }_{0}$, when there are *N* elementary source configurations with directivity functions $D_{n}\left(\theta ,\varphi_{0}\right)$, where n=1,2,…,N, the coefficients $b_{n}$ can be found by solving the following matrix equation to match the observed directivity function $D\left(\theta ,\varphi_{0}\right)$:

(5) Gb=d,

where $b_{N\times 1}$ is column vector of unknown coefficients, $b={\left[b_{1},\ldots ,b_{N}\right]}^{T}$; $d_{K\times 1}$ is a column vector of known data, $d={\left[D\left(\theta_{1},\varphi_{0}\right),\ldots ,D\left(\theta_{K},\varphi_{0}\right)\right]}^{T}$. Here, *K* represents the number of the θ-samples within a specified range $\left[\theta_{1},\theta_{2}\right]$, and $G_{K\times N}$ is a matrix constructed from the directivity functions of the elementary source configurations, with elements $G_{kn}=D_{n}\left(\theta_{k},\varphi_{0}\right)$, k=1,2,…,K. Assuming the sampling resolution of θ is sufficiently high to provide K≥N, the least-squares solution to Equation (5) is:

(6) $$\hat{b}={\left(G^{T}G\right)}^{-1}G^{T}d,$$

yielding the corresponding $\hat{d}=G\hat{b}$. If the agreement between $\hat{d}$ and d is not satisfactory, the number of the elementary source configurations should be increased, N⟼N+1, and the steps in Equations (5) and (6) need to be repeated. It is important to note that for different values of $\varphi_{0}$, the source configurations may vary, affecting both the coefficients $\hat{b}$ and even the number *N*.

## 3. Selection of Elementary Source Configurations

In the traditional ESM, the number of equivalent sources required is $Sk^{2}$, where k is the reference wave number, and S is the scattering surface area [7]. However, the method proposed in this paper only requires three basic point source configurations for high-precision modeling, which significantly reduces the number of equivalent sources compared with the traditional ESM method. This leads to a substantial reduction in computational load and time. Taking a 1 $m^{2}$ flat plate as an example, Table 3 shows a comparison of the number of equivalent sources required by the traditional ESM and the method presented in this paper at three frequencies in air. The method proposed in this paper selects $Y_{0}^{0}$, $Y_{1}^{0}$, and $Y_{1}^{1}$ at 1000 Hz; $Y_{1}^{0}$, $Y_{1}^{1}$, and $Y_{2}^{0}$ at 1500 Hz; and $Y_{1}^{1}$, $Y_{2}^{0}$, and $Y_{2}^{1}$ at 200 Hz.

The selection of elementary source configurations largely determines the accuracy and precision of the reconstruction results. Sometimes, improper selection of elementary source configurations can affect the accuracy and precision of the reconstruction results, even leading to the appearance of several or a large number of outliers in the results. In comparison, three elementary source configurations (N=3) are typically sufficient for high-precision modeling of a given simple directivity function. However, in rare cases where the radiation patterns are highly complex or when greater accuracy is needed for large elevation angles, additional elementary configurations may be required. In such cases, the matrix **G** may become ill-conditioned, and solving Equation (6) could require the use of a pseudo-inverse, which imposes specific requirements on the selection of elementary source configurations.

**Figure 1:**
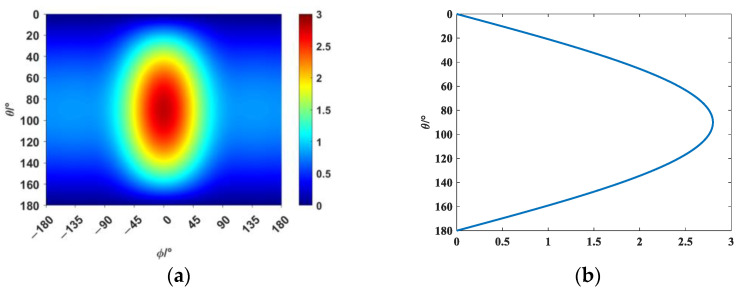
Schematic diagram of the directivity function D. (**a**) 2D image of the imaginary component of the directivity function. (**b**) Absolute value of the directivity function along the source plane φ = 0.

Therefore, this paper explores the general principles for selecting elementary source configurations by reconstructing the following directivity function shown in Figure 1:

(7) $$D\left(\theta ,\varphi \right)=2i\left(0.4+\cos^{2}\left(\varphi /2\right)\right)\left(0.25\cos\left(2\varphi \right)+0.75\right)\sin\theta$$

The three combinations formed by the four spherical harmonic functions $Y_{0}^{0}$, $Y_{1}^{0}$, $Y_{1}^{1}$, and $Y_{2}^{1}$ are shown in Table 4, and the Spearman rank correlation coefficients of these four spherical harmonic functions with the directivity function *D* are presented in Table 5. The explanation of the magnitude of the correlation coefficient is provided in Table 6.

**Figure 2:**
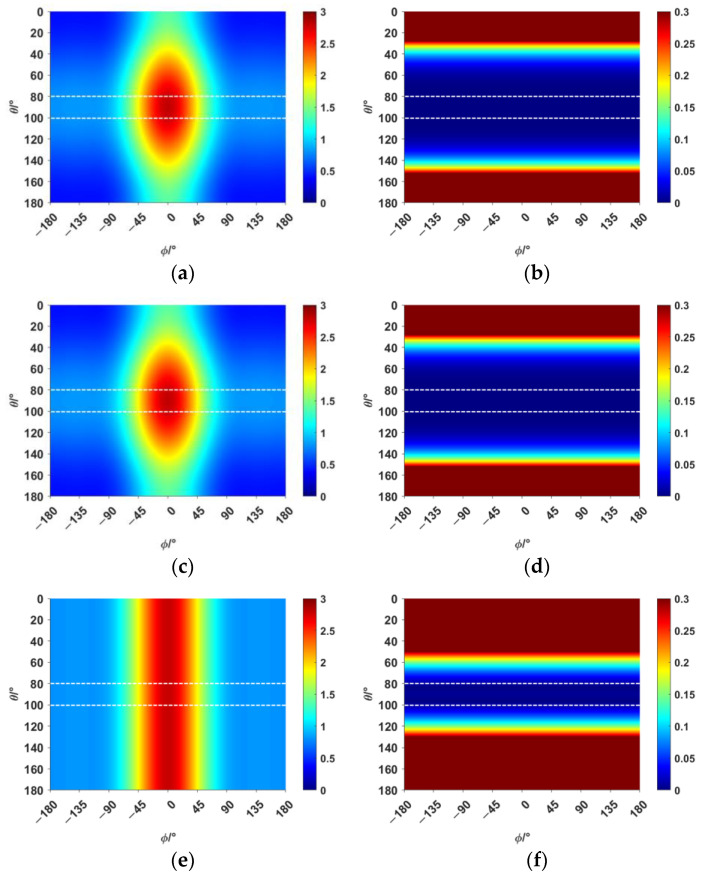
Narrow-angle reconstruction of the directivity function *D* using different combinations and the reconstruction errors ($NRMSE=\left|D-D_{0}\right|/\left|D_{0}\right|$, where $D_{0}$ is the original value and *D* is the reconstructed value). (**a**) Reconstruction using Combination 1. (**b**) Error after reconstruction using Combination 1. (**c**) Reconstruction using Combination 2. (**d**) Error after reconstruction using Combination 2. (**e**) Reconstruction using Combination 3. (**f**) Error after reconstruction using Combination 3.

The narrow-angle reconstruction of the directivity function D is performed by Combination 1, Combination 2, and Combination 3, respectively. The results are shown in Figure 2 (the narrow-angle region is enclosed by dashed lines):

In Figure 2, the reconstructed results of Combination 1 and Combination 2 show high accuracy and precision, with relatively small errors, both within and outside the narrow-angle region, compared with the original directivity function *D*. However, the reconstructed results of Combination 3 exhibit high accuracy only within the narrow-angle region, while the errors outside of the narrow-angle region are significantly larger. The elementary source configuration coefficients of each combination and the comparison of the absolute values before and after reconstruction for each combination are shown in Table 7. The computation time for each combination is shown in Table 8.

As shown in Table 5, the spherical harmonic function $Y_{1}^{1}$ has a strong correlation with the directivity function *D*. This characteristic is also reflected in Table 7. In Combination 1, the dominant contribution comes from the spherical harmonic function $Y_{1}^{1}$, while the contributions of the other two terms can be neglected. In Combination 2, the spherical harmonic function $Y_{1}^{1}$ alone is sufficient to achieve accuracy and error precision comparable to that of Combination 1. The errors in both Combination 1 and Combination 2 are within 0.1% in the narrow-angle region, which can be considered negligible. When the angle reaches 40°, the maximum error is still within 5%. Combination 3, which lacks the spherical harmonic function $Y_{1}^{1}$, only achieves high accuracy in the narrow-angle region. The error in this region is within 1%, but the error precision in the narrow-angle region is not as good as in Combination 1 and Combination 2. When the angle reaches 20°, the error exceeds 5%. As shown in Table 8, selecting the appropriate basic source configuration can reduce the computational load and save time.

Based on the above, it can be concluded that when selecting elementary source configurations, those that exhibit a strong correlation with the directivity function should be prioritized. This approach allows for achieving high accuracy and precision with as few elementary source configurations as possible. If elementary source configurations with no or weak correlation to the directivity function are selected, it not only increases unnecessary computational load but also leads to large errors and even leads to the occurrence of outliers during the least squares solution process.

## 4. Propeller Radiated Noise from Unmanned Underwater Vehicle

### 4.1. Noise Measurement Test

The UUV propeller radiated noise was tested on the lake. The experiments were carried out at the Thousand Islet Lake experimental station on 10 September 2023, where the average water depth is 60 m, and the hydrological conditions are superior.

**Figure 3:**
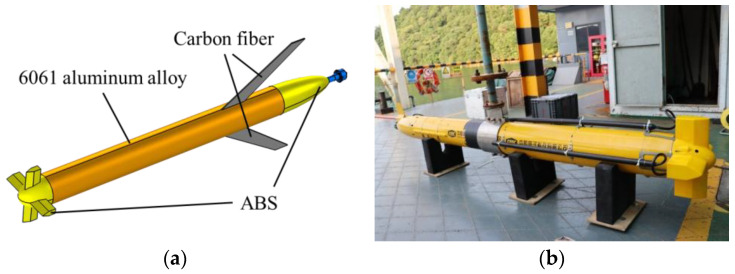
Unmanned Underwater Vehicles. (**a**) Schematic diagram of the model. (**b**) Photograph of the actual object.

**Figure 4:**
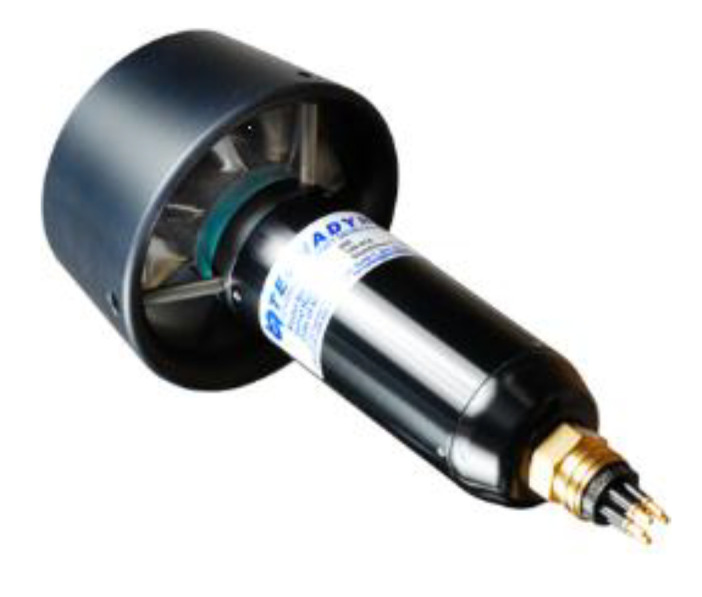
Model 260 propeller.

The experimental subject was designed by Tianjin University, with a shell thickness of 8.5 mm, and constructed using 6061 aluminum alloy. The bow and stern were made of ABS, while the wings were made of carbon fiber. The UUV consists of four compartments from the bow to the stern, with lengths of 385 mm, 495 mm, 620 mm, and 495 mm, respectively. The cabin has a diameter of 221 mm. The actual model of the UUV is shown in Figure 3, and the propeller used is Model 260, manufactured by Tecnadyne, located in San Diego, CA, USA, as shown in Figure 4.

**Figure 5:**
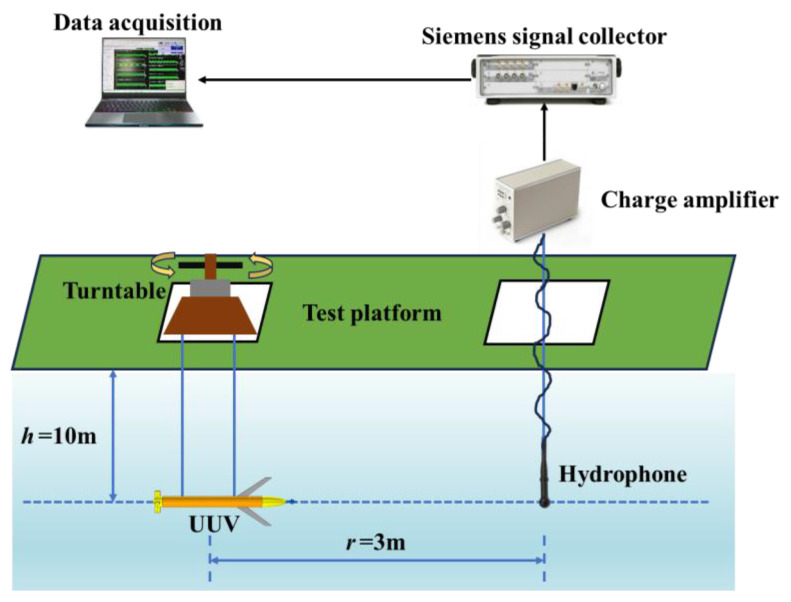
Schematic diagram of the test equipment layout.

The experimental setup is shown in Figure 5. The distance between the hydrophone and the geometric center of the UUV is *r* = 3 m. A horizontal suspension method is used, with the geometric center of both the UUV and the receiving hydrophone located on the same depth line at a depth of *h* = 10 m below the water surface. The test object is connected to the turntable via a flange, and the turntable enables rotation within a range of 0° to 360° to capture the radiated sound field at different angles.

**Figure 6:**
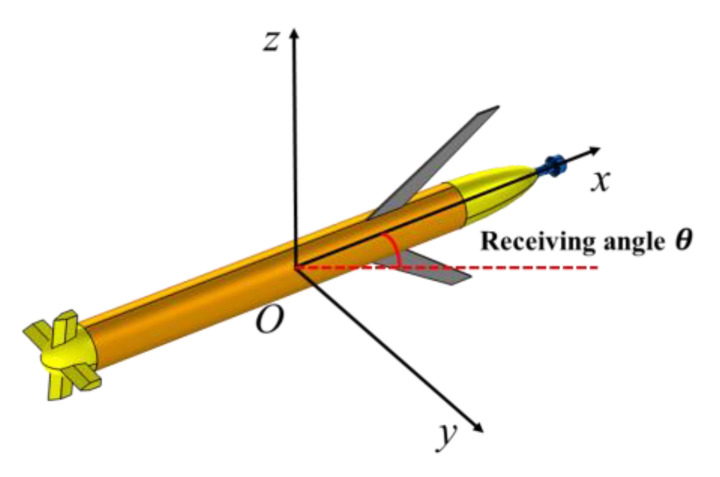
Schematic diagram of the receiving angle.

The propeller-radiated noise of the UUV exhibits directivity characteristics. Therefore, the radiated noise was measured within a receiving angle range of ±30° at a rotational speed of 1000 RPM. The reception angle of the tested UUV is shown in Figure 6.

### 4.2. Equivalence of the Measurement Results

**Figure 7:**
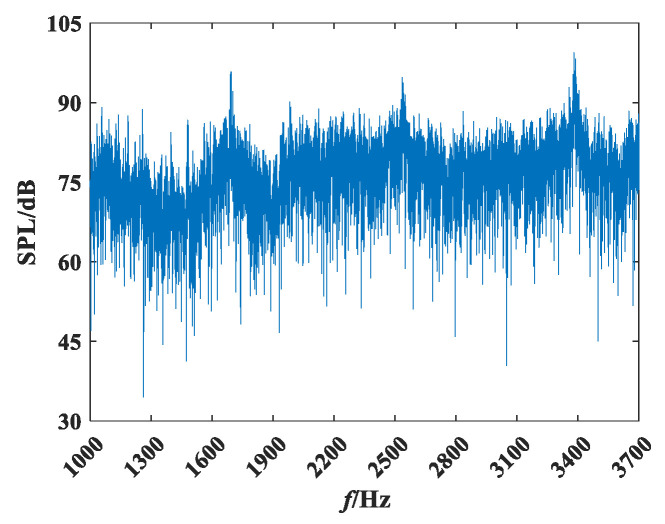
Frequency spectrum of propeller radiated noise at 0°.

The distance from the hydrophone to the target is 3 m. To satisfy the far-field condition $R\gg max\left\{\lambda ,d_{max},{d_{max}}^{2}/\lambda \right\}$, the reconstruction frequency must be above 1500 Hz. As shown in Figure 7, peaks occur at 1700 Hz, 2500 Hz, and 3400 Hz. Therefore, narrow-angle and wide-angle equivalence and error analyses were conducted for these three frequency points (where $NRMSE=\left(p-p_{0}\right)/p_{0}$, with $p_{0}$ represents the measured value, and p represents the reconstructed value).

Due to the complexity of the directional function in the test results, multiple suitable elementary source configurations were selected from the 13 spherical harmonic functions in Table 9. In Table 9, Combination 1 serves as a reference, while Combinations 2, 3, and 4 correspond to the reconstruction combinations for 1700 Hz, 2500 Hz, and 3400 Hz, respectively. Combinations 2, 3, and 4 were selected using the method from Section 3, choosing elementary source configurations that are strongly correlated with the directional function ($k_{0}d_{max}=0.1$).

**Figure 8:**
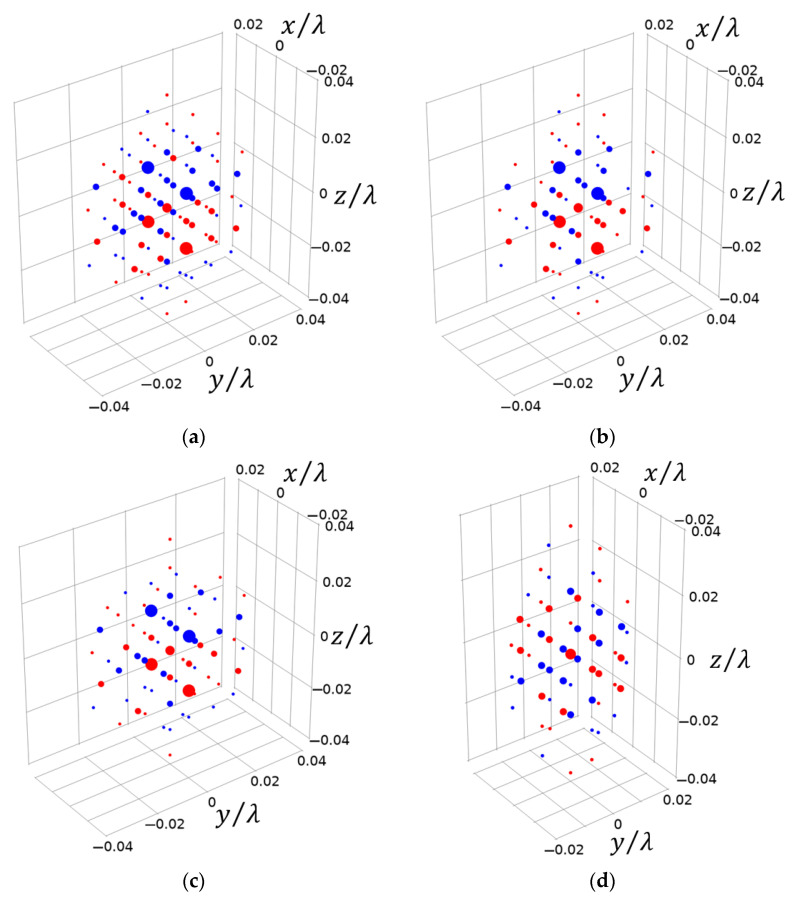
The equivalent source position distributions of the four combinations. (**a**) The equivalent source distribution of Combination 1. (**b**) The equivalent source distribution of Combination 2. (**c**) The equivalent source distribution of Combination 3. (**d**) The equivalent source distribution of Combination 4. The size of the circle represents the magnitude of the source. Different colors depict opposite source phases.

**Figure 9:**
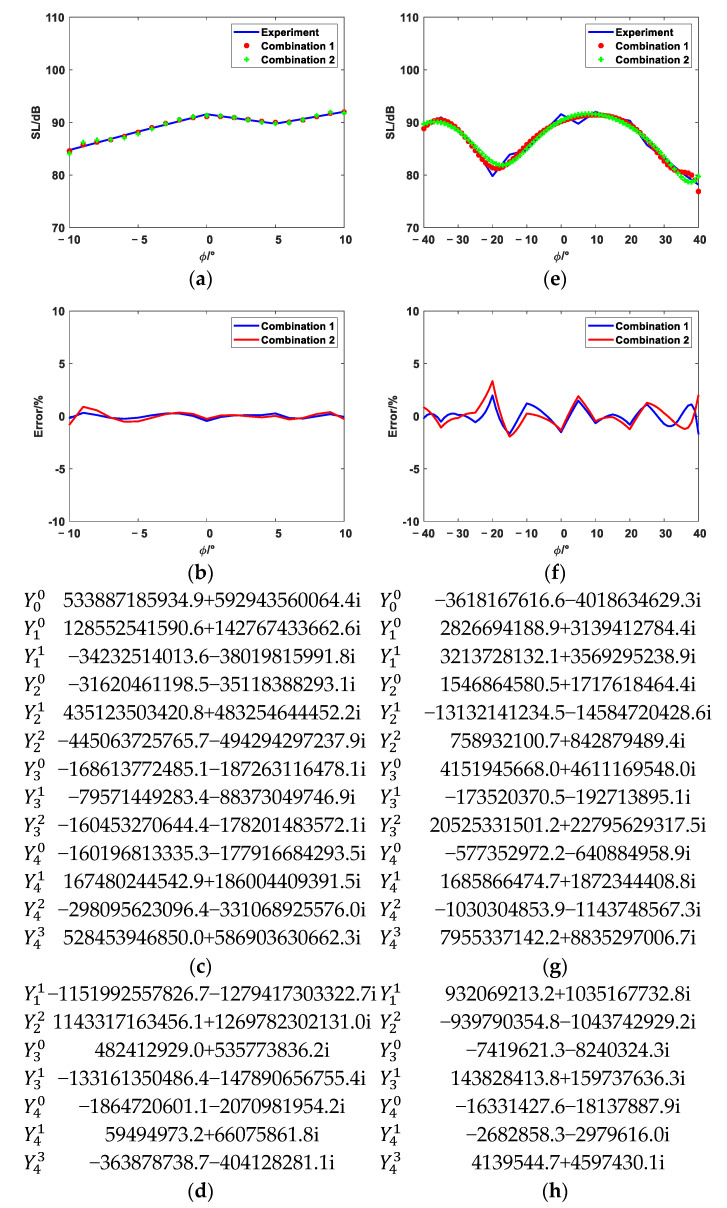
The 1700 Hz reconstruction comparison and error. (**a**) Narrow-angle reconstruction comparison diagram. (**b**) Narrow-angle reconstruction error. (**c**) Narrow-angle reconstruction coefficients of Combination 1 (**d**) Narrow-angle reconstruction coefficients of Combination 2. (**e**) Wide-angle reconstruction comparison diagram. (**f**) Wide-angle reconstruction error. (**g**) Wide-angle reconstruction coefficients of Combination 1. (**h**) Wide-angle reconstruction coefficients of Combination 2.

**Figure 10:**
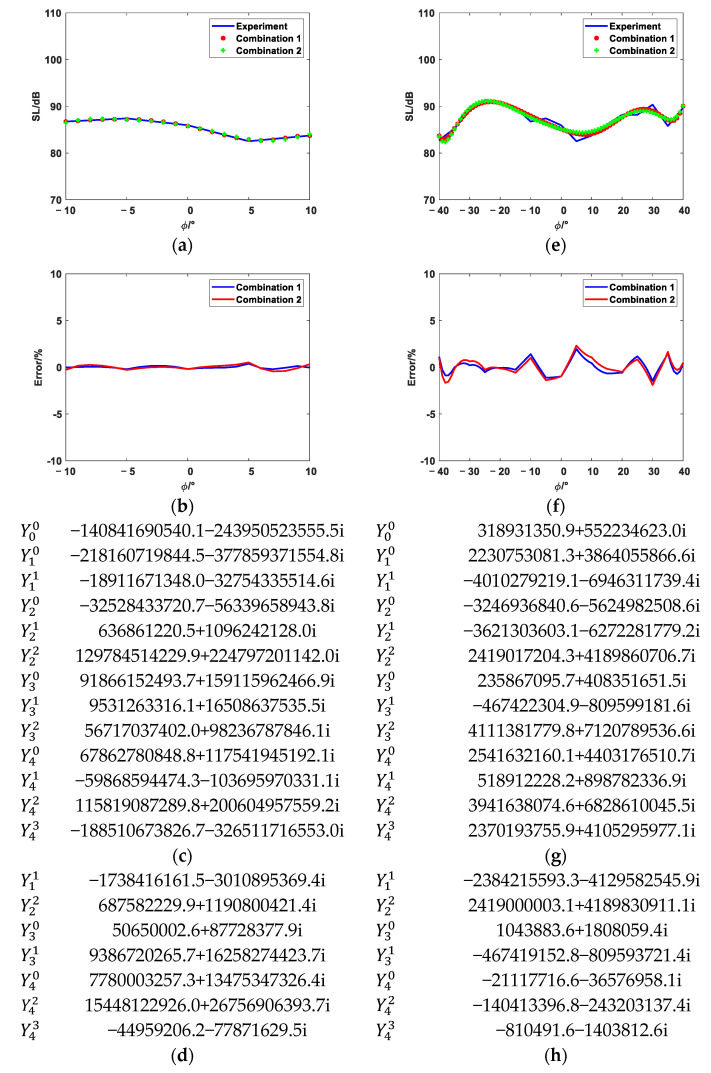
The 2500 Hz reconstruction comparison and error. (**a**) Narrow-angle reconstruction comparison diagram. (**b**) Narrow-angle reconstruction error. (**c**) Narrow-angle reconstruction coefficients of Combination 1 (**d**) Narrow-angle reconstruction coefficients of Combination 3. (**e**) Wide-angle reconstruction comparison diagram. (**f**) Wide-angle reconstruction error. (**g**) Wide-angle reconstruction coefficients of Combination 1. (**h**) Wide-angle reconstruction coefficients of Combination 3.

**Figure 11:**
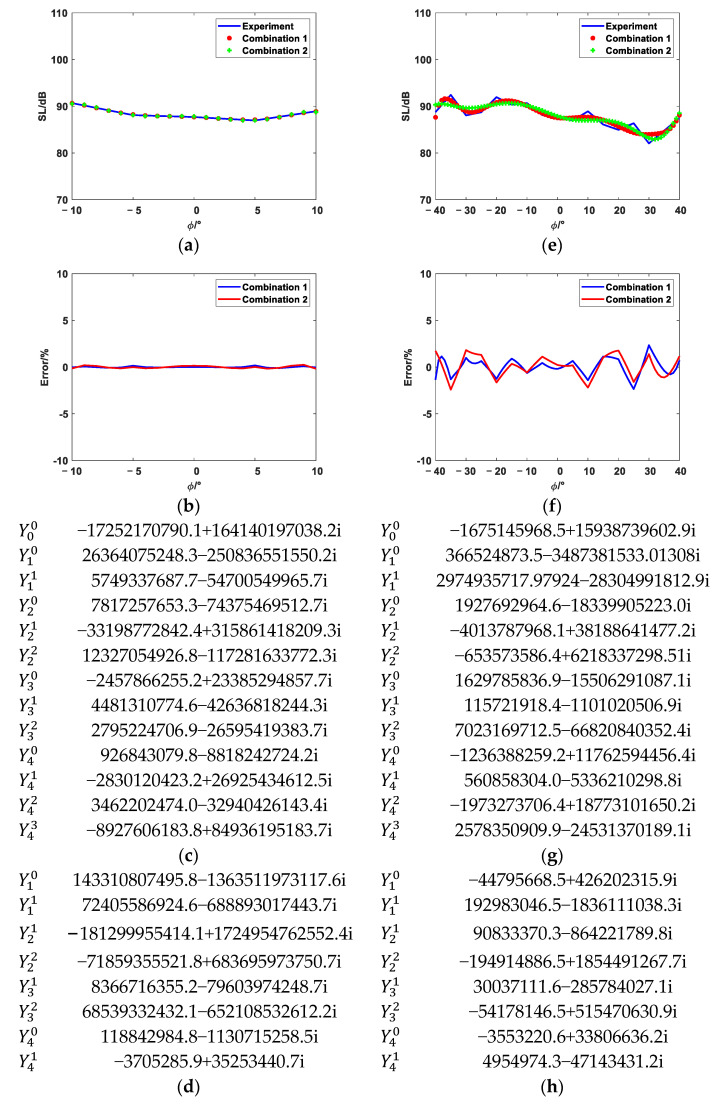
The 3400 Hz reconstruction comparison and error. (**a**) Narrow-angle reconstruction comparison diagram. (**b**) Narrow-angle reconstruction error. (**c**) Narrow-angle reconstruction coefficients of Combination 1 (**d**) Narrow-angle reconstruction coefficients of Combination 4. (**e**) Wide-angle reconstruction comparison diagram. (**f**) Wide-angle reconstruction error. (**g**) Wide-angle reconstruction coefficients of Combination 1. (**h**) Wide-angle reconstruction coefficients of Combination 4.

The equivalent source position distributions of Combination 1, Combination 2, Combination 3, and Combination 4 are shown in Figure 8. Figure 9, Figure 10 and Figure 11 show the reconstruction comparison and errors at frequencies of 1700 Hz, 2500 Hz, and 3400 Hz, respectively.

As shown in Figure 9, Figure 10 and Figure 11, both the narrow-angle and wide-angle reconstruction results are very close to the measurement values, with the error in the narrow-angle being particularly smaller. The maximum error for the narrow-angle does not exceed 1%, and the maximum error for the wide-angle does not exceed 4%, both of which are within an acceptable range. As the angular range increases, even with a 20° increase in the wide-angle range, the reconstruction error remains within 4%. The errors in Combinations 2, 3, and 4 are generally consistent with Combination 1. However, Combinations 2, 3, and 4 achieve the same reconstruction effect as Combination 1 with fewer elementary source configurations. This demonstrates that the method proposed in this paper can effectively select suitable elementary source configurations, achieving higher reconstruction accuracy with fewer elementary source configurations. However, the reconstruction error increases at the peaks of these data. The more peaks there are in these data, the larger the overall error becomes. Increasing the number of elementary source configurations has little effect on the reconstruction error at the peaks.

### 4.3. Error Analysis

**Figure 12:**
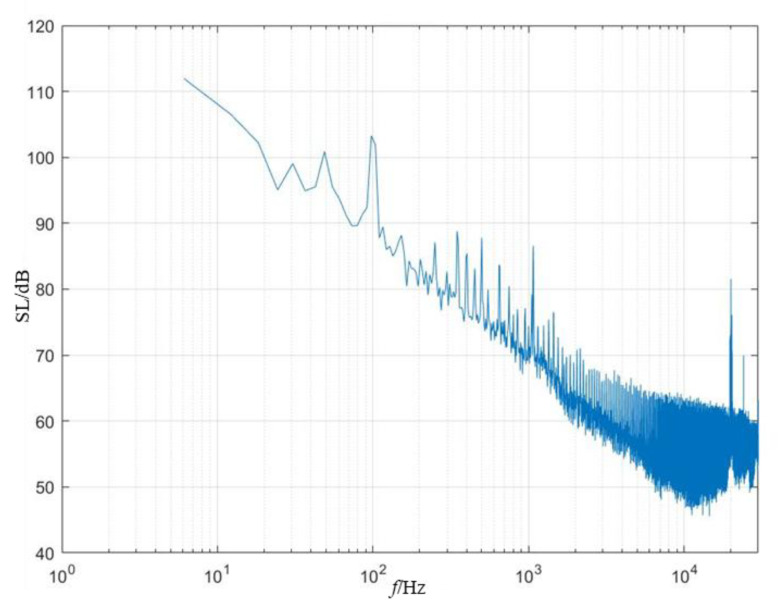
Background noise spectrum diagram.

The background noise spectrum of the experimental site is shown in Figure 12.

As seen in Figure 12, the background noise of the experimental site is relatively low, between 1 kHz and 10 kHz, with a maximum of approximately 80 dB, which meets the requirements for a low background noise environment for testing.

These test data are not perfectly left-right symmetrical. This is because the UUV was manually rotated during the test, so the rotation is not uniform. Additionally, the underwater state of the UUV was not clearly defined. Sometimes, after completing the rotation and returning to the original position, the UUV does not fully return to the exact starting point, so the 0° position does not perfectly correspond to the stern. This may lead to the test results not being entirely representative of these data within the propeller’s ±30° range, with some angular deviation. Additionally, factors such as the hydrophone possibly moving underwater during testing and whether the signal becomes distorted after amplification by the power amplifier will all affect the test results.

## 5. Conclusions

This paper discusses the general principles for selecting elementary source configurations by reconstructing the constructed directivity function. By introducing correlation coefficients, a method for selecting elementary source configurations is proposed, making the selection process no longer blind or repetitive. This method is then applied to the reconstruction of the propeller-radiated noise from a UUV, and the results verify that the method demonstrates good accuracy. The following conclusions are drawn:

When selecting elementary source configurations, those with a strong correlation to the directivity function should be chosen. Selecting elementary source configurations with no or weak correlation not only increases unnecessary computational load but also causes significant errors, even leading to outliers.

The reconstruction of the propeller radiated noise from the UUVs demonstrates that the method has good precision. It can achieve higher reconstruction accuracy with fewer elementary source configurations. The maximum error for narrow angles does not exceed 1%, and the maximum error for wide angles does not exceed 5%. When the wide-angle range is increased by 20°, the reconstruction error of the method does not increase.

The method proposed in this paper is designed for far-field reconstruction. When a larger angular range is required, more basic source configurations are needed, and the reconstruction accuracy decreases. The reconstruction error increases at the peak values in these data. Increasing the number of basic source configurations has little effect on the reconstruction error at the peak, which is an area that needs further improvement in the future.

## Figures and Tables

**Table 1:** The elementary source configurations correspond to the first three orders of spherical harmonic functions.

Spherical Harmonic Functions	Elementary Source Configurations
$Y_{0}^{0}$	Monopole
$Y_{1}^{0}$	z-axis dipole
$Y_{1}^{1}$	x-axis dipole+y-axis dipole
$Y_{2}^{0}$	z-axis tripole
$Y_{2}^{1}$	x=0 plane quadrupole+y=0 plane quadrupole
$Y_{2}^{2}$	x-axis tripole+y-axis tripole+z=0 plane quadrupole

**Table 2:** The θ-angle range and applicable scenarios for narrow-angle PE and wide-angle PE.

PE	θ-Angle Range	Applicable Scenarios
Narrow-angle	$\theta \in \left[80^{\circ },100^{\circ }\right]$	Underwater communication system, directional speakers, ultrasonic flaw detector, etc.
Wide-angle	$\theta \in \left[60^{\circ },120^{\circ }\right]$	Acoustic positioning system, aircraft radiation noise, propeller radiation noise, etc.

**Table 3:** Comparison of the Number of Equivalent Sources.

Frequency	Traditional Equivalent Source Method	Method Presented in This Paper
1000 Hz	322	7
1500 Hz	725	9
2000 Hz	1289	15

**Table 4:** Three groups of elementary source configuration combinations.

Spherical Harmonic Functions	Elementary Source Configurations	Combination 1	Combination 2	Combination 3
$Y_{0}^{0}$	Monopole	√		√
$Y_{1}^{0}$	z-axis dipole	√		√
$Y_{1}^{1}$	x-axis dipole+y-axis dipole	√	√	
$Y_{2}^{1}$	x=0 plane quadrupole+ y=0 plane quadrupole			√

**Table 5:** The correlation coefficient between the spherical harmonic functions and the directivity function *D*.

Spherical Harmonic Functions	Correlation Coefficient
$Y_{0}^{0}$	NaN
$Y_{1}^{0}$	−0.00878
$Y_{1}^{1}$	0.99991
$Y_{2}^{1}$	−0.00878

**Table 6:** Criteria for evaluating the correlation coefficient.

Correlation	Negative	Positive
No correlation	−0.09 to 0.0	0.0 to 0.09
Weak correlation	−0.3 to −0.1	0.1 to 0.3
Moderate correlation	−0.5 to −0.3	0.3 to 0.5
Strong correlation	−1.0 to −0.5	0.5 to 1.0

**Table 7:** Comparison of the coefficients of the elementary source configurations and the absolute values before and after reconstruction for each combination on the φ=0 plane.

Combinations	Elementary Source Configurations	Coefficients	Absolute Values Before and After Reconstruction at φ = 0
Combination 1	$Y_{0}^{0}$	0.0000 − 0.0709i	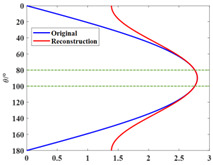
	$Y_{1}^{0}$	0.0000 − 4.3859 × 10^−15^i	
	$Y_{1}^{1}$	0.0000 + 8.1623i	
Combination 2	$Y_{1}^{1}$	0.0000 + 8.1041i	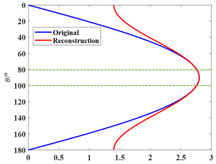
Combination 3	$Y_{0}^{0}$	0.0000 + 9.8704i	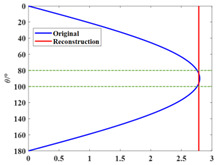
	$Y_{1}^{0}$	0.0000 − 3.1311 × 10^−11^i	
	$Y_{2}^{1}$	0.0000 + 2.0000 × 10^−11^i	

**Table 8:** The time spent on the computation for each combination.

	Combination 1	Combination 2	Combination 3
Computation time	5.47 s	3.08 s	5.53 s

**Table 9:** Four groups of elementary source configuration combinations.

Spherical Harmonic Functions	Combination 1	Combination 2	Combination 3	Combination 4
$Y_{0}^{0}$	√			
$Y_{1}^{0}$	√			√
$Y_{1}^{1}$	√	√	√	√
$Y_{2}^{0}$	√			
$Y_{2}^{1}$	√			√
$Y_{2}^{2}$	√	√	√	√
$Y_{3}^{0}$	√	√	√	
$Y_{3}^{1}$	√	√	√	√
$Y_{3}^{2}$	√			√
$Y_{4}^{0}$	√	√	√	√
$Y_{4}^{1}$	√	√		√
$Y_{4}^{2}$	√		√	
$Y_{4}^{3}$	√	√	√	

## Data Availability

All evaluated data are presented in this paper in graphical form. Original measurement data of this study are available upon request from the corresponding author.
